# The French EVAL-PLH cohort of persons with polyhandicap

**DOI:** 10.1038/s41598-022-16596-3

**Published:** 2022-07-22

**Authors:** Ilyes Hamouda, Marie-Christine Rousseau, Any Beltran Anzola, Marie-Anastasie Aim, Thierry Billette de Villemeur, Pascal Auquier, Karine Baumstarck, the EVAL-PLH group, Thierry Billette de Villemeur, Marie-Christine Rousseau, Sherezad Khaldi-Cherif, Kim Maincent, Agnès Felce, Karine Baumstarck, Pascal Auquier, Lionel Dany, Any Beltran, Ilyes Hamouda, Marie-Anastasie Aim, Narjess Boutalbi, Isabelle Kemlin, Julie Roger, Patrick Julien, Ponha Heng, Daniel Willocq, Maria Valkov, Stéphane Pietra, Stéphane Lenormand, Katia Lind

**Affiliations:** 1 EA 3279, CEReSS - Research Centre on Health Services and Quality of Life, Faculté des Sciences Médicale et Paramédicales, Aix Marseille University, 27, Boulevard Jean-Moulin, 13385 Marseille Cedex 05, France; 2 Fédération des Hôpitaux de Polyhandicap et Multihandicap, San Salvadour Hospital, University Hospital of Paris, 4312 Rte de l’Almanarre, 83400 Hyères, France; 3 UR 849, LPS - Social Psychology Laboratory, Aix-Marseille University, 29 Av. Robert Schuman, 13621 Aix-en-Provence, France; 4 Clinical Research Group GRC ConCer-LD, Sorbonne University and Pierre-et-Marie-Curie University, 4, Place Jussieu, 75005 Paris, France; 5 Department of Neuropediatrics, Armand-Trousseau Hospital, 26 Av. du Dr Arnold Netter, 75012 Paris, France; 6 Hôpital Trousseau, Service de Neuropédiatrie - Pathologie du développement, APHP, Paris, France; 7 Hôpital San Salvadour, Hyères, APHP, Paris, France; 8 Union Générale Caisse Assurance Maladie (UGECAM), Ile de France, France; 9 Comité d’Éducation et de Soins Auprès des Personnes Polyhandicapées, CESAP, Paris, France; 10 Hôpital d’Hendaye, APHP, Hendaye, France; 11 Hôpital de La Roche Guyon, La Roche-Guyon, France

**Keywords:** Medical research, Neurology

## Abstract

Polyhandicap is characterized by a combination of profound intellectual disability and serious motor deficit, resulting in the extreme restriction of autonomy and communication. The aim of the EVAL-PLH (EVALuation PoLyHandicap) study is to identify the impact of socioeconomic, environmental, and epidemiological determinants on the health status of the persons with polyhandicap and the daily lives of their caregivers. EVAL-PLH is a prospective cohort study. The study involved persons with severe polyhandicap (who were cared for at reeducation centers, residential facilities, and one specialized pediatric/neurological department of a university hospital), their familial caregivers and the institutional caregivers. Data collection included sociodemographics, heath status, and psychocomportemental information. Data have been collected at 2 points (2015-2016 and 2020-2021). The French EVAL-PLH cohort is the first cohort study focusing on persons with polyhandicap, their families, and the health care workers caring for them. The sustainability of the device is essential to assist patients, families, clinicians, and health decision-making authorities in the optimization of care management.

## Introduction

Profound intellectual and multiple disabilities as a chronic complex disability condition, manifest as a combination of profound intellectual disability and serious motor deficit, resulting in the extreme restriction of autonomy and communication. When the disorder affects an immature brain, the term polyhandicap is used^1^. This definition was adopted by the French scientific community and by French law (French Law no. 89-798, October 27, 1989, Health Policy of Disability Care). Polyhandicap, considered a group of profound intellectual and multiple disabilities or cerebral palsy, causes multiple forms of disability due to an affect arising during the brain’s early development^2^. In European countries, the prevalence of cases is estimated to be approximately 1 per 1000^3–5^, depending on the definition used. Robust and recent epidemiologic data are lacking (prevalence, incidence).

People with polyhandicap require specific medical and technical care and preventive actions that include f the social and educational dimensions necessary to offer them a coherent, adapted, and integrated life. Some countries have moved toward extensive deinstitutionalization, including of the most dependent patients, transferring the burden of care to families. In contrast, in France, persons with more severe chronic conditions are very rarely deinstitutionalized. The French health system is supposed to offer a graduated response allowing these patients to benefit from three main care management modalities: specialized rehabilitation centers, residential facilities, and home care^6^. The specialized rehabilitation centers offer a high level of medical and paramedical physical rehabilitation, while residential facilities offer a high level of psychosocial education and a lower level of medical care. Home care refers to the care of patients (adults and children) living at home; the family may benefit from specific nursing and medical care for the patient. The care management of this chronic complex disability differs by country, depending on both the specificities of the associated health care system and the related societal views^7^. However, the health care pathway is not always optimal and may lack flexibility due to insufficient geographic coverage, and some adults may remain in pediatric facilities due to a lack of space in centers for adults^8^; thus, more robust data are needed on the adequacy of care management for French patients with polyhandicap^8^.

Throughout their lives, patients with polyhandicap need continuous health and educational support and human and technical assistance^9,10^. Institutional or familial caregivers who care for persons with polyhandicap must be able to cope with completely dependent patients with dramatic clinical conditions. The psychological distress and health deterioration of institutional caregivers have been previously described^11–15^, including those of health care workers in institutions for persons with disabilities^16^. Similarly, families, particularly parents, who are constantly confronted with stressful situations from the first years of the life of the child, reported a considerable impact of polyhandicap on the social, psychological, and physical aspects of their lives^17^. More data are needed to strengthen these observations and better assist clinicians and health decision-making authorities in offering more appropriate interventions.

The optimization of care management for persons with polyhandicap and their caregivers is possible with increasing knowledge of the condition’s clinical characterization and care trajectories and the experiences, beliefs and representations of caregivers. From this perspective, and to improve the knowledge of this population of patients, their specific needs and the impacts on their caregivers, we implemented a French prospective cohort study, the EVAL-PLH (EVALuation PoLyHandicap) study, which was designed to include the assessment of 3 different populations: persons with polyhandicap, familial caregivers, and institutional caregivers. The general aim of the study was to identify the impact of socioeconomic, environmental, and epidemiological determinants on the health status of persons with polyhandicap and the daily lives of their (familial and institutional) caregivers. The specific objectives were to (1) describe the evolution over time of the health status of persons with polyhandicap; (2) describe the evolution over time of the life experiences of families; (3) describe the evolution over time of the life experiences of institutional caregivers; (4) describe the health care pathways of persons with polyhandicap throughout their lives; and (5) estimate the incidence, prevalence, and survival of persons with polyhandicap in France. No similar evaluation exists in France or in other countries. The main findings were disseminated by means of many international publications^2,7–10,16–19^. This paper details the major elements of the EVAL-PLH study protocol.

## Methods/design

### Design and funding

This is a prospective cohort study. Thus far, data have been collected at 2 points. The first part of the study was organized between 2015 and 2016, and the second part was organized between 2020 and 2021. Before the second part, the study design was a cross-sectional design. The cohort design beaun in the second part. This is the reason why we submitted the protocol study at this time. At its initiation, the EVAL-PLH study was supported by the French PREPS (Programme de recherche sur la performance du système de soins, Direction Générale de l’Organisation des Soins, 2013) and the French Institut National de la Santé et de la Recherche Médicale (INSERM, 2013).

The study is registered in the ClinicalTrials.gov database (Trial registration: NCT02400528; registered 27/03/2015). The sponsorship was represented by Assistance Publique, Hôpitaux de Marseille, France; its role was to control the appropriateness of the ethical and legal considerations. All methods were carried out in accordance with relevant French guidelines and regulations. The research was approved by the French ethics committee (name: Comité de Protection des Personnes Sud Méditerranée V; postal address: CHU·HOPITAL DE CIMIEZ, Nice, France; website: https://www.cpp-sud-mediterranee-v.fr/; approbation date: 20/10/2014; reference number: 2014-A00953-44; check-in number: 14.041). All experiments were performed in accordance with relevant guidelines and regulations. The research was performed in accordance with the Declaration of Helsinki. A written consent form was obtained from each participant and/or their legal guardians.

### Settings

The EVAL-PLH cohort was formed on 01/03/2015, and it involved persons with severe polyhandicap who were cared for at the following centers: four reeducation centers, eight residential facilities (the French Comité d'Études, d'Education et de Soins Auprès des Personnes Polyhandicapées Association (CESAP)), and one specialized pediatric/neurological department of a university hospital (Service de Neuropédiatrie, UPMC, Hôpital Trousseau, Assistance Publique Hôpitaux de Paris, France). The four reeducation centers receive inpatients needing significant medical care for long durations (many days, months, and sometimes years) through conventional hospitalization stays (specialized reeducation centers). Residential facilities receive inpatients and outpatients needing less intensive medical care. The specialized pediatric/neurological department receives children with their families cared for and managed at home. These families, supported by human (nurses, physiotherapists, etc) and technical (wheelchair, specific bed, and other devices) assistance at home, benefit from an annual follow-up at the hospital.

### Populations

Three different populations were eligible: (1) persons with severe polyhandicap; (2) familial caregivers of the included persons; and (3) institutional caregivers of the included persons.

The selection criteria were as follows:

1. Persons with severe polyhandicap are defined as persons experiencing a combination of motor deficiency (tetraparesis, hemiparesis, paraparesis, extra pyramidal syndrome, cerebellar syndrome, neuromuscular problems) and profound intellectual impairment (intelligence quotient < 40) associated with everyday life dependence (Functional Independence Measure < 55) and restricted mobility (Gross Motor Function Scale III, IV and V).
2. Familial caregivers of the included persons who were aged above 18 years; were the referent person who were contacted for any medical, administrative, and social issues; and agreed to participate. Parents, siblings, and other familial members were eligible. In the second wave, due to the low participation rate (< 5%) of participants who were not parents, only parents were included.
3. Institutional caregivers of the included persons who were aged above 18 years; were an institutional referent caregiver of at least one patient who was included in the cohort; and agreed to participate (institutional referent caregiver was designated by the health care team for each patient with polyhandicap; he or she was the resource person who had to coordinate various issues concerning the patient, such as care management, family contact, and administrative and social issues).

The exclusion criteria for persons with polyhandicap were age at inclusion < 3 years, intelligence quotient ≥ 40, dependence with a Functional Independence Measure ≥ 55, or an I/II level at the Gross Motor Function Scale. The exclusion criterion for caregivers was refusal to participate. A written consent form was obtained from each participant.

### General procedure

The initiation of each wave of the cohort study relies on the collaboration between a specialist in neuropediatrics (Prof. Thierry Billette de Villemeur) and a specialist in epidemiology (Prof. Pascal Auquier). A steering committee consisting of a physician caring for persons with severe polyhandicap (Dr Marie-Christine Rousseau), an epidemiologist (Dr Karine Baumstarck), and psychologists (Tanguy Leroy, Lionel Dany) led the survey. This committee is in charge of the general organization of the study: coordination, regulatory issues, and finding solutions for any dysfunction. All study partners (the physicians and the scientific, administrative, and logistical actors) constitute the EVAL-PLH group [see Additional file 1. The EVAL-PLH group]. Data were collected from the first 2 evaluations. The first wave was organized between 2015 and 2016, the second wave was organized between 2020 and 2021, and the third wave is planned for 2025–2026.

Each wave is organized according to the following steps:

1. Persons with polyhandicap: The list of eligible patients is established by a dedicated clinical research assistant in each center and checked by the patients’ referring physicians. Clinical data were collected from the medical records. The list of eligible patients is updated at each wave (notification of deceased persons, identification of new cases, and investigation of patients who switch centers). The data are collected by a (permanent or not) doctor of the center. The doctor was experienced with persons with polyhandicap, and was encouraged to question the healthcare team in charge of the person.
2. Familial caregivers: A maximum of 2 familial referents are systematically identified for each included person. Contact information is updated whenever possible. A survey booklet is sent by mail to each familial referent. To optimize participation, a prepaid return envelope addressed to the coordination team is included to the mail. In the case of nonresponses, a recall system is planned.
3. Institutional caregivers: The members of the steering committee hold meetings with the institutional caregivers in each center to explain the objectives and modalities of the study. A self-report survey booklet is given to each reference health care worker volunteering to participate. No specification is given concerning the place where they had to fill out the booklet (at the hospital or not). To optimize participation, a recall system is planned.

### Data collection

Various sources of data are used. An additional details file shows this in more detail [see Additional file 2, Data collection details]

1. For persons with polyhandicap, the data are collected from the medical records: sociodemographics, severity and stability, associated handicaps, comorbidities, neurodevelopmental patterns, medical devices, rehabilitation procedures, and medical treatments.
2. For familial caregivers, the data are gathered into a booklet, including the following: sociodemographics, nature of their relationship with the person with polyhandicap, marital status, number of children, educational level, occupational status, financial status, presence of the person with polyhandicap at home, personal health data (chronic diseases; use of health resources), psycho-behavioral data (anxiety, mood disorders, coping, quality of life, and burden), and specific information about the social environment and satisfaction with health care.
3. For institutional caregivers, the data are gathered into a booklet, including the following: sociodemographics, marital status, number of children, educational level, financial situation, notion of a handicapped person living at home, personal health data, professional situation (job categories, work schedule, experience in disability care), and psycho-behavioral data (anxiety, mood disorders, coping, quality of life, and burn-out).

### Data quality, data monitoring, and statistical aspects

The data quality is ensured by the dedicated clinical research assistant of the study (AB). The data entry plan and the data monitoring plan have been approved by the operational committee (including the clinical research assistant (AB), the statistician (IH), the referent epidemiologist (KB), and the physician experienced in polyhandicap (MCR)). The sponsor (Assistance Publique des Hôpitaux de Marseille) conducted an auditing trial at the end of the 1st wave including the number of participants and consent collection. The second auditing trial is planned in 2022 for the second wave. No independent audit was planned. The database is securitally stored in the dedicated department of the sponsor (Epidemiologie department, the Service d’Epidémiologie et Economie de la Santé, Assistance Publique des Hôpitaux de Marseille, France. Prof. Pascal Auquier (PA) is responsible for the department and the referent epidemiologist for EVAL-PLH is Dr Karine Baumstarck (KB). The security of the database and the confidentiality are based on the appropriate French guideline (French National Data Protection Committee (CNIL) published new reference regarding data processing in health research to adapt the existing framework to the EU General Data Protection Regulation (GDPR)). The statistical analysis plan (SAP) was developed in 2015 by the statistician and the referent epidemiologist, and was updated in 2019. Analyses of data will be based on the following steps: (1) Cross-sectional study of the populations (persons with polyhandicap, familial caregivers, and institutional caregivers) assessed at the 2nd wave: descriptive data and identification of determinants (health, psychobehavioral profiles, quality of life); and (2) Longitudinal study for the persons assessed in the first 2 waves: analyses of changes over time in clinical data, psychobehavioral data, quality of life, identification of predictors, and description of care pathways. The SAP is available upon reasonable request.

### Ethics approval and consent to participate

All methods were carried out in accordance with relevant French guidelines and French regulations. The research was approved by the French ethics committee (name: Comité de Protection des Personnes Sud Méditerranée V; postal address: CHU·HOPITAL DE CIMIEZ, Nice, France; website: https://www.cpp-sud-mediterranee-v.fr/; approbation date: 20/10/2014; reference number: 2014-A00953-44; check-in number: 14.041). All experiments were performed in accordance with relevant guidelines and regulations. The research has been performed in accordance with the Declaration of Helsinki. A written consent form was obtained from each participant and/or their legal guardians.

### Consent for publication

A written consent form was obtained from each participant and/or their legal guardians.

## Results

At this time, only data from the first wave (2015–2016) of the study were explored. A total of 875 persons with polyhandicap, 440 familial caregivers (390 parents), and 362 institutional caregivers were included. These findings were provided across more than ten international publications. The main findings highlighted the following: (1) The variety of clinical statuses: for the first time, the health status of the persons with polyhandicap, aged 3–70 years, was described in terms of etiologies, comorbidities, associated handicaps, behavioral disorders, treatments and devices, severity and stability^9,18^; (2) The evolving nature of the health status across the lifespan: for the first time, we showed the complex and specific needs of persons with polyhandicap in terms of care at every period of life, in particular the transition process from childhood to adulthood^10^; (3) The adequacy of the care management: we showed that the adequacy of care management (in terms of care structures, nature of care provided) from objective indicators (such as the health severity and the age) seems less satisfactory than the perceived adequacy of the physicians^8^; (4) The considerable impact on the quality of life for parents^19^ and for health care workers caring for individuals with severe polyhandicap^16^. Readers may refer to the respective references for more details.

The data from the first wave were also used to validate two new major tools for the assessment of individuals with polyhandicap. First, the polyhandicap severity scale^7^ provides the first reliable and valid measure of the health severity status for children and adults with polyhandicap that can be easily applied in research and clinical practice. Second, the PolyQoL questionnaire is a short proxy-reported questionnaire assessing the quality of life of persons with polyhandicap (Hamouda I. Quality of life of polyhandicapped persons: initial validation of the PolyQoL. SOFMER 2021; October, Lille, France).

The enrollment of participants in the second wave of the study is now closed, and we will proceed to the quality control of the data. The analysis plan is fixed according to the different objectives. We will begin the data validation in 2022.

Additional funding has been received for ancillary studies. The POLYMIME study and the POLYRENE project were both supported by the French National Organization of Solidarity for Autonomy (Caisse Nationale de Solidarité pour l’Autonomie (CNSA)) in 2019 and 2021, respectively. POLYMIME (Familial impact of POLYhandicap: MIxed MEthod approach) aimed to more specifically explore the disappointments, expectations, and wishes of the parents of individuals with polyhandicap. The quantitative approach is entirely included in the EVAL-PLH study. The qualitative approach will allow us to better understand the daily lives of the families. Interviews (from 27 to 54 according to speech saturation) were conducted by phone (due to restrictions related to the COVID-19 pandemic) with parents of persons with polyhandicap, audio-recorded and transcribed by a researcher trained in ongoing qualitative studies^20^. The audiotapes were also made available to the second researcher involved in the triangulation (Aim MA et al., Lived experience and family impact of polyhandicap: preliminary results of interviews with parents. SOFMER 2021; October, Lille, France). Ttriangulation^21^ was carried out in order to enhance credibility of the findings from the 2 approaches^22^.

The aim of POLYRENE (POLYhandicap: the French REsearch Network) is to expand the initial community of the EVAL-PLH to include underrepresented populations and to improve the visibility of the cohort. The project begins in 2022.

## Discussion

EVAL-PLH is the first large cohort study focusing on polyhandicap with a strict definition of the patients included. It is a unique project in France, Europe and the world. Some specific aspects can be highlighted.

First, the longitudinal approach provides more valid information than cross-sectional studies. In the scientific literature, the previous studies only relied on small sample sizes and heterogeneous populations, focused on narrow objectives, and used retrospective or cross-sectional designs. Longitudinal studies allow us to better understand the observed phenomena and explore the dynamics and modeling sequences between them.

Second, the inclusion of persons who receive care in various care modalities may provide a robust picture of the global situation. Better knowledge of the health care pathways may optimize the care management of patients with polyhandicap according to their specific needs in terms of age, dependency degree, severity status, and families’ wishes.

Third, the assessment of familial caregivers’ experience is currently recognized as essential. There is a vast body of literature assessing caregiving in various chronic/severe diseases (such as cancer^23,24^, mental health diseases^25^, various neurologic diseases^26,27^, and cerebral palsy^28–30^), but very few studies have assessed the impact of polyhandicap on caregivers^17,19^. Consideration of the experiences and concerns of families would strongly assist clinicians and health decision-making authorities in offering appropriate interventions.

Fourth, the importance of assessing the experiences of health care workers is also acknowledged. These caregivers work in a specific context that includes frequent physical tasks due to the complete physical dependence of the patients, the difficult and challenging personal histories of the patients and their distressed families, and restricted feedback and recognition of the care they provide due to the limitations of communication with the patient and the distance of the families. Moreover, residential facilities and specialized reeducation centers for persons with polyhandicap have a high absenteeism rate. A better understanding of this experience may assist health facility managers and care teams in taking appropriate targeted actions.

### Limitations and solutions

The representativeness of the sample is questionable. We can assume that the specialized rehabilitation centers included in the study provide a high representativeness of patients with polyhandicap who are cared for in specialized rehabilitation centres. Approximately 70% of hospital beds dedicated to patients with polyhandicap in France were located in these centers^8^. However, the representativeness of the residential facilities is more questionable. The recruitment of patients cared for in residential facilities was exclusively based on voluntary participation. Concerning the persons whose care is managed at home, they all came from a single department, which reduced the representativeness. In the third wave, the inclusion of patients cared for at home and in medicosocial structures must be emphasized. This is what the POLYRENE project anticipates.

The interpretation of the data collected during 2020–2021 should be made taking account the unquestioned impact of the COVID-19 pandemic. It was a very special time for persons with polyhandicap and their caregivers, both familial, but also institutional, resulting huge amounts of stress: virulence and lack of knowledge about the disease (in particular for the vulnerable populations), mobility restrictions, and mask wearing. The findings should definitely be analyzed taking into account this major event.

The first analysis showed us which variables and which scales were not met or incorrectly fulfilled. In the next waves, the data collection could be improved. Some questions could be reformulated, some tools could be substituted (for example, preferring a validated tool to assess anxiety-depression instead of single score), and some aspects, not yet assessed, could be added. The final decision always relies on the balance between validity and acceptability.

**Figure 1:**
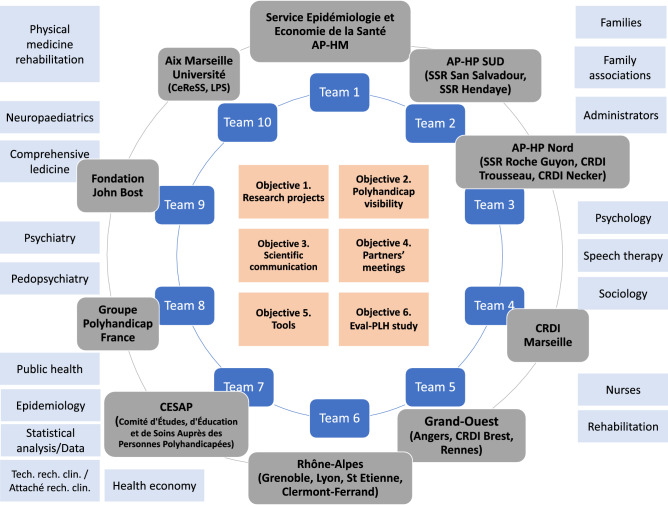
The POLYRENE project.

The representativeness of the members of the steering committee should be discussed. The First Steering Committee only included medical specialists and psychologists. It is now essential to include professionals from a wide range of disciplines, such as sociologists, economists, public health professionals, social workers, and caregiver association representatives. This is planned in the POLYRENE project (Fig. 1).

The visibility of the EVAL-PLH study is insufficient. New communication tools should be used to better state the importance of the device. The POLYRENE project includes the availability of a website, newsletter distribution, scientific finding dissemination, dedicated days for workshops and webinars, and the planning and organization of research projects.

## Conclusion

The French EVAL-PLH cohort is the first cohort study focusing on persons with polyhandicap, their families, and the health care workers caring for them. The sustainability of the device is essential to assist patients, families, clinicians and health decision-making authorities in the optimization of care management.

### Trial status

The study is registered in the ClinicalTrials.gov database (Trial registration: NCT02400528; registered 27/03/2015). The EVAL-PLH cohort was formed on 01/03/2015, it is an open cohort. Protocol version: Version 2 of 18/09/2014

## Supplementary Information

Supplementary Information.

## Data Availability

The access to the full protocol, participant-level data, datasets, and statistical code can be made available from the corresponding author upon reasonable request.
