# Supplementary material for: The French EVAL-PLH cohort of persons with polyhandicap
Source: Sci Rep. 2022 Jul 22;12:12512. doi: 10.1038/s41598-022-16596-3 (PMC9305042; doi:10.1038/s41598-022-16596-3)
Supplement: Supplementary file 1 — Supplementary Information. [file 41598_2022_16596_MOESM1_ESM.pdf]

Additional file 1. The EVAL-PLH group

The EVAL-PLH group 2015-2016

The French Polyhandicap Group includes for the first wave, the following individuals: Thierry Billette de Villemeur (lead author, thierry.billette@aphp.fr, Hôpital Trousseau, Service de Neuropédiatrie - Pathologie du développement, Paris, APHP, France), Marie-Christine Rousseau (Hôpital San Salvadour, Hyères, APHP, France), Sherezad Khaldi-Cherif (Union Générale Caisse Assurance Maladie (UGECAM), Ile de France, France), Catherine Brisse (Comité d'Études, d'Éducation et de Soins Auprès des Personnes Polyhandicapées, Paris, France), Agnès Felce (Hôpital d'Hendaye, Hendaye, France), Karine Baumstarck (EA 3279, Self-perceived Health Assessment Research Unit, School of Medicine, Aix Marseille Université, France), Pascal Auquier (EA 3279, Self-perceived Health Assessment Research Unit, School of Medicine, Aix Marseille Université, France), Tanguy Leroy (EA 4163, Laboratoire GRePS, Université Lyon 2, France), Cécile Freihuber (Hôpital Trousseau, Paris, France), Sofiane Amalou (Hôpital Trousseau, Paris, France), Julie Bonheur (Hôpital Trousseau, Paris, France), Stéphanie Valence (Hôpital Trousseau, Paris, France), Marie-Christine Nougues (Hôpital Trousseau, Paris, France), Laurent Luciani (Le Poujal, CESAP, Thiais, France), Jean-Pierre Nouet (Le Poujal, CESAP, Thiais, France), Catherine Coiffier (Sessad, CESAP, Saint-Maurice, France), Sophie Mathieu (Hôpital de La Roche Guyon, La Roche-Guyon, France), Moustafa Ardati (Hôpital de La Roche Guyon, La Roche-Guyon, France), Delphine Héron (Hôpital Trousseau, Paris, France), Alexandra Afenjar (Hôpital Trousseau, Paris, France), Kim Maincent (Hôpital Trousseau, Paris, France), Diana Rodriguez (Hôpital Trousseau, Paris, France), Diana Doummar (Hôpital Trousseau, Paris, France), Marie-Laure Moutard (Hôpital Trousseau, Paris, France), Daniel Willocq (Hôpital San Salvadour, Hyères, France), Maria Valkov (Hôpital San Salvadour, Hyères, France), Julie Teulade (Hôpital San Salvadour, Hyères, France), Stéphane Pietra (Hôpital San Salvadour, Hyères, France), Stéphane Lenormand (Hôpital d'Hendaye, Hendaye, APHP, France), Elizabeth Grimont (Hôpital San Salvadour, Hyères, APHP, France).

The EVAL-PLH group 2020-2021

The French Polyhandicap Group includes for the second wave, the following individuals: Thierry Billette de Villemeur (lead author, thierry.billette@aphp.fr, Hôpital Trousseau, Service de Neuropédiatrie - Pathologie du développement, Paris, APHP, France), Marie-Christine

Rousseau (Hôpital San Salvadour, Hyères, APHP, France), Sherezad Khaldi-Cherif (Union Générale Caisse Assurance Maladie (UGECAM), Ile de France, France), Kim Maincent (Comité d'Études, d'Éducation et de Soins Auprès des Personnes Polyhandicapées, Paris, France), Agnès Felce (Hôpital d'Hendaye, Hendaye, APHP, France), Karine Baumstarck (EA 3279, Self-perceived Health Assessment Research Unit, School of Medicine, Aix Marseille Université, France), Pascal Auquier (EA 3279, Self-perceived Health Assessment Research Unit, School of Medicine, Aix Marseille Université, France), Lionel Dany (LPS Laboratory of social Psychology, Aix Marseille Université, France), Any Beltran (EA 3279, Self-perceived Health Assessment Research Unit, School of Medicine, Aix Marseille Université, France), Ilyes Hamouda (EA 3279, Self-perceived Health Assessment Research Unit, School of Medicine, Aix Marseille Université, France), Marie-Anastasie Aim (LPS Laboratory of social Psychology, Aix Marseille Université, France), Narjess Boutalbi (CESAP, France), Isabelle Kemlin (CESAP, France), Julie Roger (CESAP, France), Patrick Julien (CESAP, France), Ponha Heng (Hôpital de La Roche Guyon, Daniel Willocq (Hôpital San Salvadour, Hyères, APHP, France), Maria Valkov (Hôpital San Salvadour, Hyères, APHP, France), Stéphane Pietra (Hôpital San Salvadour, Hyères, APHP, France), Stéphane Lenormand (Hôpital d'Hendaye, Hendaye, APHP, France), Katia Lind (Union Générale Caisse Assurance Maladie (UGECAM), Ile de France).

Additional file 2. Data collection details

For persons with polyhandicap, the data are collected from the medical records:

1) Socio demographic data: age, gender, place in the sibling (elder or not), structure of care management (specialized rehabilitation center, residential facility, home care), and health care pathway from birth to assessment.

2) Etiologies of polyhandicap: unknown etiology, known etiology (central nervous system malformations, neurometabolic and neurogenetic encephalopathy, epileptic encephalopathy, pre/perinatal and post-natal causes).

3) Health status:

- Global health severity: i. severe for patients who meet all the following criteria: motor handicap (paraparesia or tetraparesia and/or extrapyramidal syndrome and/or severe general hypotonia), IQ <25, FIM <=20, and GMFCS IV and V; ii. less severe for patients who do not meet these criteria,
- Global health stability: i. unstable for patients who meet at least one of the following criteria: recurrent pulmonary infections (>=5/yrs), drug-resistant epilepsia (>=4 seizures/month); ii. stable for patients who do not meet any of these criteria,
- Associated handicaps: i) severe motor handicaps: tetraparesia, paraparesia, hemiplegia; ii) other neurologic handicaps: movement disorders, severe dystonia, severe hypotonia, extrapyramidal syndrome, ataxia; iii) neuro-sensorial handicaps: visual impairment (partial/complete blindness) and hearing impairment (partial/complete deafness); iv) behavioral disorders (including intermittent screaming, and/or agitation and/or stereotypies and/or intermittent crying and/or self-aggressivity and/or hetero-aggressivity), v) sleep disturbance (short sleep, night wake up, and difficulties falling asleep).
- Co-morbidities: epilepsia (yes/no, previous status epilepticus, drug-resistant disease), orthopedic (scoliosis, limb deformations, limb fractures, hip luxation, previous arthrodesis and/or other previous orthopedic surgery), pulmonary (pulmonary recurrent infections, aspiration syndrome), digestive (fecal impaction, gastro esophageal reflux, and drooling), urinary (recurrent urinary tract infections-at least once a year and urinal retention), cutaneous (bedsores, pressure sore), chronic pain, chronic diseases (at least one of the following diseases: vascular stroke, myocardial infarction, diabetes, and/or cancer), antibiotic resistant bacteria.

- Neurodevelopmental patterns: i. autonomy: seven facets of autonomy were scored by the referent physician from 1 (worse) to 7 (best autonomy/developmental degree) for the following domains: visual contact, oral language, postural ability, grasping ability, moving ability, cleanliness, and feeding ability; ii. general neuro-developmental status: it was assessed using an adapted version of the Brunet-Lézine scale. This scale was available for infants up to 24-month old (1). In the present study, only the 4 developmental domains (language, posture-motor abilities, coordination, and sociability) were used (2). All scores ranged from 0 to 24 months.

4) Medical devices and rehabilitation procedures management:

- Medical devices: at least one, number, and type (invasive mechanical ventilation, non-invasive mechanical ventilation, tracheotomia, nasogastric tube, gastrostomy, permanent urinary probe, cerebrospinal fluid derivation, and central venous catheter).
- Rehabilitation procedures: bed without orthesis, limb orthesis, verticalization device.

For familial caregivers, the data are gathered into a booklet

1) Sociodemographics and general information: age, gender, nature of the relationship with the polyhandicapped person, marital status (not single/single), in couple with the other parent of the polyhandicapped person, number of children living at home and notion of another handicapped person living at home, educational level, occupational status (worker/not worker), self-perceived financial status, importance of the presence of the polyhandicapped person at home (<7/>= 7 nights/month).
2) Health and presence and nature of chronic diseases; hospitalization episode during the last 2 years; other health resources use during the past 3 months (anxiety and/or stress medications, psychological support, alternative medicines).
3) Anxiety-mood disorders assessed using a score ranged from 1 (absence) to 10 (very important).
4) Coping assessed using the Brief Coping Orientation to Problems Experienced Scale (Brief-COPE) (3), exploring 4 dimensions that include social support, problem solving, avoidance, and positive thinking (4). Scores ranged from 0 to 100. High scores reflect a high tendency to implement the corresponding coping strategies.
5) Quality of life assessed using the World Health Organization Quality of Life (WHOQOL-BREF) questionnaire which is a generic questionnaire used worldwide. It describes four

domains: physical health, psychological health, social relationships, and environment. All scores range between 0 and 100, with higher scores indicating a better QoL. French norms are available for three domains (5).

6) Burden: i) specific ad hoc questions: number of children living at home, hours of daily caregiving, and frequency of getting up during the night; ii) self-perceived burden using the Caregiver Reaction Assessment scale (CRA)(6), a 24-item questionnaire describing five subscales: self-esteem (seven items), family support (five items), financial impact (three items), planning impact (five items), and health impact (four items). Scores ranged from 1 to 5. For the self-esteem subscale, a higher score indicated that caregiving had a more positive impact; a score >=4 was used to indicate a high positive impact. For the other four subscales, higher scores indicated that caregiving had a more negative impact; a score >=3 was used to indicate a high negative impact. All the scores were linearized on a 0-100 scale.

7) Specific questions are proposed regarding: i) the social environment: family relationship preservation, presence of the PLH individual during family celebrations, existence of a social network related to the PLH/not related to the PLH, PLH associative community implication. ii) the parents' healthcare satisfaction: medical information related to the PLH individual, global management information quality, quality of care provided to the PLH individual, family caregivers services provided.

For institutional caregivers, the data are gathered into a booklet

1) Sociodemographics and general information: age, gender, marital status (not single/single), children, educational level, self-perceived financial status, notion of a handicapped person living at home, and chronic disease(s);

2) Professional situation: job categories (technical: nurses, physiatrists, psychomotricians; basic care: nurse aid, education care: educators), work schedule (full time, part time), years of experience in PLH care, years of experience in the present center, notion of specific professional formation for PLH, and nature of the center (reeducation center, residential facility);

3) Anxiety-mood disorders assessed using a score ranged from 1 (absence) to 10 (very important).

4) Coping strategies assessed using the Brief Coping Orientation to Problems Experienced Scale (Brief-COPE) (see above).

5) Quality of life assessed using the World Health Organization Quality of Life (WHOQOL-BREF) questionnaire (see above).

6) Occupational burn out assessed using the Malash Burnout Inventory (MBI) recognized as a valid and reliable tool of occupational burnout syndrome assessment. The MBI provides a global score. From the global score, three levels of burn out were defined: high, moderate or low (7).

References :

1. Josse D. Brunet-Lézine Révisé: Echelle de développement psychomoteur de la première enfance. Éd. et applications psychologiques. 1997.
2. Rousseau MC, Leroy T, Auquier P, Billette De Villemeur T. The use of the brunet lezine scale for the cognitive and motor assessment of patients with profound and multiple disabilities. Ann Phys Rehabil Med. 1 sept 2015;58:e132-3.
3. Assessing coping strategies: A theoretically based approach. - PsycNET [Internet]. [cité 8 févr 2022]. Disponible sur: https://doi.apa.org/doiLanding?doi=10.1037%2F0022-3514.56.2.267
4. Baumstarck K, Alessandrini M, Hamidou Z, Auquier P, Leroy T, Boyer L. Assessment of coping: a new french four-factor structure of the brief COPE inventory. Health Qual Life Outcomes. 11 janv 2017;15(1):8.
5. Baumann C, Erpelding M-L, Régat S, Collin J-F, Briançon S. The WHOQOL-BREF questionnaire: French adult population norms for the physical health, psychological health and social relationship dimensions. Rev Epidemiol Sante Publique. févr 2010;58(1):33-9.
6. Given CW, Given B, Stommel M, Collins C, King S, Franklin S. The caregiver reaction assessment (CRA) for caregivers to persons with chronic physical and mental impairments. Res Nurs Health. août 1992;15(4):271-83.
7. Maslach C, Jackson S, Leiter M. The Maslach Burnout Inventory Manual. In: Evaluating Stress: A Book of Resources. 1997. p. 191-218.
